# Protease-activated receptor-1 impairs host defense in murine pneumococcal pneumonia: a controlled laboratory study

**DOI:** 10.1186/cc11910

**Published:** 2012-12-27

**Authors:** Marcel Schouten, Cornelis van't Veer, Joris JTH Roelofs, Marcel Levi, Tom van der Poll

**Affiliations:** 1 Center for Experimental and Molecular Medicine (CEMM), Academic Medical Center, University of Amsterdam, Meibergdreef 9, 1105 AZ Amsterdam, The Netherlands; 2 Center for Infection and Immunity Amsterdam (CINIMA), Academic Medical Center, University of Amsterdam, Meibergdreef 9, 1105 AZ Amsterdam, The Netherlands; 3 Department of Pathology, Academic Medical Center, University of Amsterdam, Meibergdreef 9, 1105 AZ Amsterdam, The Netherlands; 4 Department of Internal Medicine, Academic Medical Center, University of Amsterdam, Meibergdreef 9, 1105 AZ Amsterdam, The Netherlands; 5 Division of Infectious Diseases; Academic Medical Center, University of Amsterdam, Meibergdreef 9, 1105 AZ Amsterdam, The Netherlands

## Abstract

**Introduction:**

*Streptococcus pneumoniae *is the most common causative pathogen in community-acquired pneumonia. Protease-activated receptor-1 (PAR-1) is expressed by multiple cell types present in the lungs and can be activated by various proteases generated during acute inflammation. The cellular effect of PAR-1 activation partially depends on the specific protease involved. We here determined the role of PAR-1 in the host response during murine pneumococcal pneumonia.

**Methods:**

Wild-type (WT) and PAR-1 knockout (KO) mice were infected intranasally with viable *S. pneumoniae *and observed in a survival study or euthanized at 6, 24 or 48 hours of infection.

**Results:**

PAR-1 KO mice had a better survival early after infection compared to WT mice. Moreover, PAR-1 KO mice had lower bacterial loads in lungs and blood at 24 hours and in spleen and liver at 48 hours after infection. This favorable response was accompanied by lower lung histopathology scores and less neutrophil influx in PAR-1 KO mice.

**Conclusion:**

PAR-1 impairs host defense during murine pneumococcal pneumonia.

## Introduction

*Streptococcus (S.) pneumoniae *or the pneumococcus is the number one causative pathogen in community-acquired pneumonia (CAP) [1]. CAP is an important cause of sepsis: in a recent large sepsis trial 35.6% of the patients suffered from severe CAP, with the pneumococcus being the most frequent cause [2]. Worldwide *S. pneumoniae *is responsible for approximately ten million deaths annually, making pneumococcal pneumonia and sepsis a major health threat [3].

Protease-activated receptors (PARs) are G protein-coupled receptors that are abundantly expressed in the lungs [4-6]. PARs, of which four family members have been described (PAR-1 to -4), carry their own ligand: proteolytic cleavage leads to exposure of a neo-amino terminus, which serves as a ligand for the same receptor, hereby initiating transmembrane signaling. A variety of proteases can activate PARs, including several proteases involved in the coagulation system. Intriguingly, activation of PAR-1 can result in opposite cellular effects depending on the protease involved in its proteolytic cleavage: for example high concentrations of thrombin can cause barrier disruptive effects on vascular endothelium via activation of PAR-1, whereas the anticoagulant protein activated protein C (APC) exerts barrier protective and anti-inflammatory effects via the same receptor [7-9].

We here considered it of interest to investigate the effect of PAR-1 activation on the course of pneumococcal pneumonia. Thus far, data on the role of PAR-1 in severe bacterial infection are limited to studies using endotoxemia or polymicrobial peritonitis induced by cecal ligation and puncture (CLP) as models of severe sepsis. Kaneider *et al *used a pepducin-based approach to show that activation of PAR-1 is harmful during the early phases of endotoxemia and CLP-induced sepsis, but beneficial at later stages [10]. Somewhat contradicting, Niessen *et al *showed that PAR-1 is harmful during early as well as late stages of endotoxemia and sepsis induced by CLP, with a pivotal role for dendritic cell signaling [11]. We here for the first time studied the role of PAR-1 in respiratory tract infection, using our well-established clinically relevant model of pneumococcal pneumonia, comparing survival, antibacterial defense and inflammatory responses in PAR-1 knockout (KO) and normal wild-type (WT) mice. We show that in pneumococcal pneumonia, PAR-1 impairs host defense, as reflected by a reduced lethality and lower bacterial loads, lung histopathology scores and less pulmonary neutrophil influx in PAR-1 KO mice.

## Materials and methods

### Animals

Heterozygous PAR-1 KO mice on a C57Bl/6 background were purchased from The Jackson Laboratory (Bar Harbor, ME, USA) [12]. Animals were intercrossed to obtain homozygous PAR-1 KO mice. WT C57BL/6 mice were purchased from Charles River (Maastricht, the Netherlands). All experiments were approved by the Institutional Animal Care and Use Committee of the University of Amsterdam.

### Experimental infection and sample harvesting

Pneumonia was induced by intranasal inoculation with approximately 5 × 10^4 ^colony-forming units (CFU) of *S. pneumoniae *serotype 3 (American Type Culture Collection, ATCC 6303, Rockville, MD, USA) as described [13,14]. Mice were sacrificed after 6, 24 or 48 hours of infection (n = 8 per group per time point) or observed for 4 days in a survival study (n = 14 per group). On predefined time points mice were anesthetized, citrated plasma was prepared from blood drawn from the vena cava inferior and left lung homogenates were prepared as described [13,14]. Bacterial loads were determined as described [13,14]. For further measurements, homogenates were diluted 1:2 with lysis buffer (300 mM NaCl, 30 mM Tris, 2 mM MgCl_2_, 2 mM CaCl_2_, 1% (v/v) Triton X-100, pH 7.4) with protease inhibitor mix and incubated for 30 minutes on ice, followed by centrifugation at 680 g for 10 minutes. Supernatants were stored at -20ºC until analysis.

### Histology and immunohistochemistry

The right lung was fixed in 10% formalin/PBS for 24 hours and embedded in paraffin. Sections of 5 µm were cut, stained with hematoxylin and eosin (H & E) and analyzed by a pathologist who was blinded for groups as described [14]. To score lung inflammation and damage, the entire section was analyzed with respect to the following parameters: bronchitis, interstitial inflammation, edema, endothelialitis, pleuritis and thrombus formation. Each parameter was graded on a scale of 0 to 4. The total histopathological score was expressed as the sum of the scores. Granulocyte staining was performed using fluorescein isothiocyanate-labeled anti-mouse Ly-6G monoclonal antibody (mAb) (Pharmingen, San Diego, CA, USA) as described [15,16]. Ly-6G stained slides were photographed with a microscope equipped with a digital camera (Leica CTR500, Leica Microsystems, Wetzlar, Germany). Ten random pictures were taken per slide. Stained areas were analyzed with Image Pro Plus (Media Cybernetics, Bethesda, MD, USA) and expressed as percentage of the total surface area.

### Assays

Tumor necrosis factor (TNF)-α, interleukin (IL)-6, IL-10, IL-12p70, interferon (IFN)-γ and monocyte chemoattractant protein (MCP)-1 were measured by cytometric bead array (CBA) multiplex assay (BD Biosciences, San Jose, CA, USA). Macrophage inflammatory protein (MIP)-2 was measured by ELISA (R&D systems, Minneapolis, MN, USA).

### Statistical analysis

Data are expressed as box-and-whisker diagrams depicting the smallest observation, lower quartile, median, upper quartile and largest observation, as medians with interquartile ranges or as Kaplan Meier plots. Differences between groups were determined with Mann-Whitney U or log rank test where appropriate. Analyses were performed using GraphPad Prism version 4.0 (GraphPad Software, San Diego, CA, USA). *P *values less than 0.05 were considered statistically significant.

## Results

### Survival

**Figure 1:**
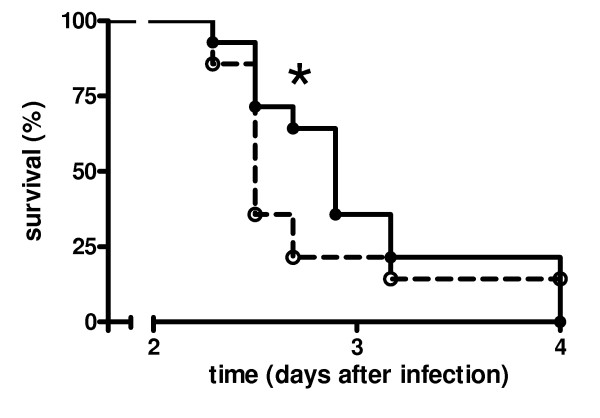
**Protease-activated receptor-1 accelerates mortality in murine pneumococcal pneumonia**. Survival of wild-type (dashed line, open symbols) and protease-activated receptor-1 knockout (solid line, closed symbols) mice in murine pneumococcal pneumonia (14 mice per group). * indicates statistical significance as compared to wild-type (*P *<0.05, log rank test).

To determine whether PAR-1 is important for outcome in pneumococcal pneumonia a survival study was performed (Figure 1). PAR-1 KO mice had a significantly delayed mortality as compared to WT mice (*P *<0.05). Median survival time was 2 days and 21 hours in PAR-1 KO mice as compared to 2 days and 12 hours in WT mice. Moreover, at 2 days and 17 hours after infection, 64% of PAR-1 KO mice was still alive, while only 21% of WT mice had survived until that time point.

### Bacterial outgrowth

**Figure 2:**
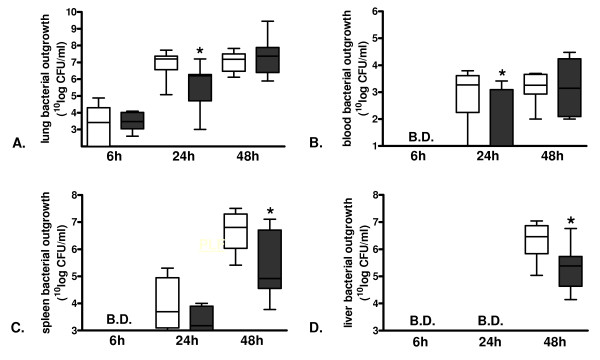
**Lower bacterial outgrowth in protease-activated receptor-1 knockout mice in lungs and blood at 24 hours and in spleen and liver at 48 hours after induction of murine pneumococcal pneumonia**. Bacterial outgrowth in **(A) **lung, **(B) **blood, **(C) **spleen and **(D) **liver 6, 24, and 48 hours after induction of pneumococcal pneumonia in wild-type (open bars) and protease-activated receptor-1 knockout (grey bars) mice. Data are expressed as box-and-whisker diagrams depicting the smallest observation, lower quartile, median, upper quartile and largest observation (eight mice per group). * indicates statistical significance as compared to wild-type (*P *<0.05, Mann-Whitney U test).

To determine whether the difference in survival between PAR-1 KO and WT mice in pneumococcal pneumonia could be attributed to a difference in antibacterial defense, we determined bacterial outgrowth 6, 24 and 48 hours in lungs, blood and distant organs (spleen, liver) (Figure 2). At 6 hours after infection, there were no differences in pulmonary bacterial loads between PAR-1 KO and WT mice (Figure 2A). At this time point, bacteria could not be detected in blood and distant organs (Figure 2B to 2D). At 24 hours, PAR-1 KO mice had markedly lower bacterial burdens in their lungs (Figure 2A) and blood (Figure 2B) with a trend toward lower levels in spleen (*P *= 0.18) (Figure 2C) as compared to WT mice. Whereas at 48 hours the differences in bacterial outgrowth in lung and blood had subsided (Figure 2A to 2B), PAR-1 KO mice had lower bacterial loads in spleen (Figure 2C) and liver (Figure 2D) as compared to WT mice.

### Inflammatory response

**Figure 3:**
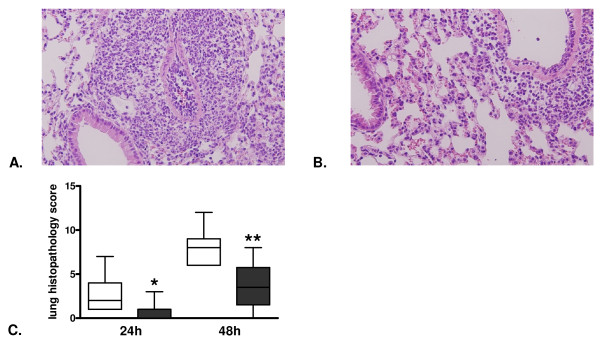
**Lower histopathology scores in protease-activated receptor-1 knockout mice in murine pneumococcal pneumonia**. Representative microphotographs of hematoxylin and eosin stained lung sections, 48 hours after induction of pneumococcal pneumonia in **(A) **wild-type and **(B) **protease-activated receptor-1 knockout mice (100 times original magnification). **(C) **Total pathology scores 24 and 48 hours after induction of pneumococcal pneumonia in wild-type (open bars) and protease-activated receptor-1 knockout mice (grey bars). Data are expressed as box-and-whisker diagrams depicting the smallest observation, lower quartile, median, upper quartile and largest observation (eight mice per group). * and ** indicate statistical significance as compared to wild-type (*P *<0.05 and *P *<0.01 respectively, Mann-Whitney U test).

**Figure 4:**
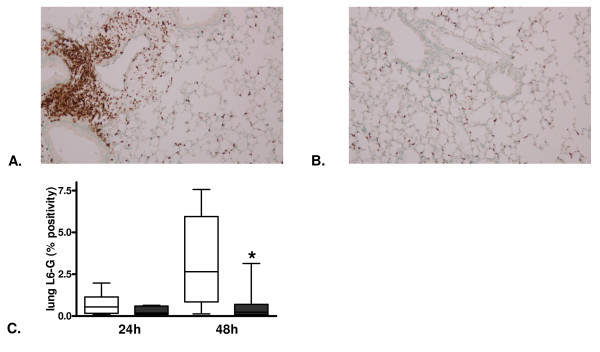
**Reduced pulmonary neutrophil influx in protease-activated receptor-1 knockout mice in later stage of murine pneumococcal pneumonia**. Representative slides of lung Ly-6G staining (brown) 24 and 48 hours after induction of pneumococcal pneumonia in **(A) **wild-type and **(B) **protease-activated receptor-1 knockout mice (200 times original magnification). **(C) **Quantitation of pulmonary Ly-6G 48 hours after induction of pneumococcal pneumonia in wild-type (open bars) and protease-activated receptor-1 knockout mice (grey bars). Data are expressed as box-and-whisker diagrams depicting the smallest observation, lower quartile, median, upper quartile and largest observation (eight mice per group). * indicates statistical significance as compared to wild-type (*P *<0.05, Mann-Whitney U test).

To investigate the impact of PAR-1 on lung pathology, we determined histopathology scores of lung tissue slides obtained 24 and 48 hours after infection. Pneumococcal pneumonia was associated with pulmonary inflammation and damage as evidenced by the occurrence of bronchitis, interstitial inflammation, edema and endothelialitis. Mean histopathological scores were lower in PAR-1 KO mice at both 24 and 48 hours after infection (Figure 3A to 3C). To obtain insight in the role of PAR-1 in neutrophil recruitment to the primary site of infection, we performed Ly-6G staining on lung sections at 24 and 48 hours after infection. While there were no significant differences at 24 hours after infection, PAR-1 KO mice showed significantly lower neutrophil numbers in lung tissue later on, as evidenced by lower Ly-6G positivity at 48 hours after infection (Figure 4A to 4C).

To further investigate the role of PAR-1 in the local inflammatory response, we determined levels of various cytokines (TNF-α, IL-6, IL-10, IL-12, IFN-γ) and chemokines (MCP-1, MIP-2) in lung homogenates at 6, 24 and 48 hours after infection (Table 1). During the first 24 hours after infection pulmonary cytokine and chemokine levels did not differ between PAR-1 KO and WT mice. At 48 hours, lung levels of TNF-α, IL-6 and IFN-γ were considerably higher in PAR-1 KO mice as compared to WT mice (*P *<0.01 to *P *<0.05), whereas pulmonary IL-10, MCP-1 and MIP-2 concentrations did not differ between groups. IL-12 remained undetectable in lung homogenates at all time points.

**Table 1:** Pulmonary cytokine and chemokine levels in wild-type (WT) and protease-activated receptor-1 (PAR-1 KO) mice 6, 24 and 48 hours after induction of pneumococcal pneumonia.

	T = 6		T = 24		T = 48	
	
	WT n = 8	PAR-1 KO n = 8	WT n = 8	PAR-1 KO n = 8	WT n = 8	PAR-1 KO n = 8
TNF-α (pg/ml)	4.45 (3.28-7.10)	5.70 (5.20-7.20)	10.5 (1.30-24.0)	13.0 (3.50-56.1)	27.3 (15.9-40.6)	175 (43.7-215)**

IL-6 (pg/ml)	7.95 (2.50-11.3)	11.6 (10.0-14.7)	227 (23.0-1418)	366 (15.5-1460)	217 (147-382)	770 (397-1642)*

IL-10 (pg/ml)	B.D.	B.D.	44.3 (34.2-66.6)	34.7 (30.5-39.3)	16.9 (10.0-24.8)	25.1 (16.3-169)

IFN-γ (pg/ml)	B.D.	B.D.	11.7 (9.30-17.4)	13.7 (3.10-34.0)	4.80 (2.90-8.85)	16.7 (11.3-23.2)*

MCP-1 (ng/ml)	0.15 (0.13-0.29)	0.19 (0.12-0.28)	8.38 (4.30-9.93)	5.28 (2.48-7.28)	3.29 (2.88-6.34)	5.69 (2.68-10.1)

MIP-2 (ng/ml)	B.D.	B.D.	6.89 (3.81-15.9)	12.6 (0.20-37.6)	1.95 (0.77-17.3)	1.14 (1.01-5.05)

Data are medians (interquartile ranges). TNF-α, tumor necrosis factor-α; IL, interleukin; MCP-1, monocyte chemotactic protein-1; MIP-2, macrophage inflammatory protein-2. B.D., below detection determined. * and ** indicate statistical significance compared to wild-type (WT) (*P *<0.05 and *P *<0.01 respectively, Mann-Whitney U test).

To investigate the role of PAR-1 in the systemic inflammatory response, we determined levels of the above mentioned cytokines in plasma (Table 2). At 6 hours after infection, cytokine levels were below detection (data not shown). At 24 hours after infection, PAR-1 KO mice had substantially lower plasma levels of TNF-α and MCP-1 (*P *<0.001) and a trend toward lower IL-6 concentrations (*P *= 0.08) when compared with WT mice. These differences had subsided at 48 hours. IL-10, IL-12 and IFN-γ levels stayed below detection throughout the course of the disease (data not shown).

**Table 2:** Plasma cytokine and chemokine levels in wild-type (WT) and protease-activated receptor-1 (PAR-1 KO) mice 24 and 48 hours after induction of pneumococcal pneumonia.

	T = 24		T = 48	
	
	WT n = 8	PAR-1 KO n = 8	WT n = 8	PAR-1 KO n = 8
TNF-α (pg/ml)	12.2 (11.4-15.4)	7.00 (4.10-9.00)**	13.7 (7.80-30.6)	17.6 (14.1-22.7)

IL-6 (pg/ml)	51.0 (8.58-92.9)	8.10 (2.50-49.8)	58.9 (49.8-210)	51.5 (41.2-69.7)

MCP-1 (ng/ml)	129 (48.6-152)	23.9 (18.7-41.6)**	75.0 (64.7-386)	151 (68.2-191)

Data are medians (interquartile ranges). TNF-α, tumor necrosis factor-α; IL-6, interleukin-6; MCP-1, monocyte chemotactic protein-1. ** indicates statistical significance compared to wild-type (WT) (*P *<0.01 respectively, Mann Whitney U test).

## Discussion

*S. pneumoniae *is a major cause of morbidity and mortality in humans and antibiotic resistance in this pathogen is increasing, which urges the need to study the host defense mechanisms that influence the outcome of pneumococcal pneumonia and sepsis [1]. In pneumonia and sepsis PARs are considered to play a pivotal role in the crosstalk between coagulation and inflammation [4]. Since data on the role of PAR-1 in severe infection are sparse and the function of PAR-1 in bacterial pneumonia and sepsis to date is unknown, we here investigated the involvement of PAR-1 in the host response to pneumococcal pneumonia. We show that PAR-1 hampers antibacterial defense, which is associated with more lung damage, more lung neutrophil influx and more systemic inflammation, altogether resulting in a higher mortality.

Previous studies examined the role of PAR-1 in endotoxemia and abdominal sepsis induced by CLP, revealing partially contradicting results [10,11,17]. Our finding that PAR-1 deficiency improves survival early in severe murine pneumococcal pneumonia is in accordance with data by Niessen *et al*, who, using a PAR-1 antagonist, showed that functional PAR-1 reduces survival in polymicrobial sepsis induced by CLP, a finding which was associated with dendritic cell-mediated sustainment of proinflammatory and procoagulant mechanisms [11]. These authors also showed that PAR-1 KO mice had a better survival in a 90% lethal dose (LD90) model of endotoxin-induced toxicity [11], a finding that differed from an earlier study demonstrating an unaltered mortality of PAR-1 KO mice after a high-dose endotoxin challenge [17]. In contrast to the studies performed by Niessen and colleagues [11], the survival benefit of PAR-1 KO mice in our study was only temporary. This does not necessarily mean there is no effect of PAR-1 deficiency in later stages of the disease but could be related to the fact that our model of severe pneumococcal pneumonia is an LD100 model as opposed to the models used by Niessen *et al *[11]. Additional studies using lower infectious doses are warranted to establish whether PAR-1 deficiency impacts on survival in less severe pneumonia. Kaneider *et al *reported an unaltered survival of PAR-1 KO mice relative to WT mice in CLP-induced sepsis [10]. However, they also showed that early treatment with a PAR-1 antagonist (at t = 0) did improve survival in CLP, whereas administration of a PAR-1 agonist at a later time point (t = 4 hours) also conveyed a survival benefit [10]. From their studies these investigators concluded that PAR-1 is detrimental in early phases of sepsis but beneficial in later phases, which could explain the absence of a net survival benefit in PAR-1 KO mice in their studies [10]. A very recent study identified matrix metalloproteinase (MMP)-1a as a PAR-1 agonist in mice; blockade of MMP-1a activity protected against CLP-induced lethality in WT but not in PAR-1 KO mice, suggesting that MMP-1 activation of PAR-1 contributes to an adverse outcome of polymicrobial abdominal sepsis [18]. Clearly, the studies on the role of PAR-1 endotoxic shock and CLP-induced sepsis are not fully consistent. We did not evaluate the effects of pharmacologic blockade of PAR-1 in pneumococcal pneumonia; such studies could reveal potential time-dependent effects of PAR-1 inhibition and the possible impact of therapeutic PAR-1 blockade in the context of concurrent antibiotic treatment.

The survival advantage of PAR-1 KO mice in our study corresponded with lower bacterial loads at various stages of the infection. In addition, PAR-1 KO mice displayed lower lung pathology scores and a reduced number of neutrophils in lung tissue. The mechanisms underlying these differences remain to be elucidated. Understanding the role of PAR-1 signaling in infection is difficult due to the multiple and in part opposite effects ascribed to this receptor. Indeed, although APC and thrombin can both activate PAR-1, APC affects the vascular endothelium in a way that clearly is distinct from thrombin signaling. Specifically, APC can exert anti-inflammatory, anti-apoptotic and vasculoprotective signals in endothelial cells via PAR-1, processes in which the endothelial protein C receptor plays a pivotal role [7,19], whereas thrombin induces vascular hyperpermeability via PAR-1 [20]. To make things more complex, activation of PAR-1 by low doses of thrombin can (like APC) result in a barrier protective effect [8], whereas a very recent investigation provided evidence that activated coagulation factor VII (FVIIa) can exert a barrier protective effect in endothelial cells via activation of PAR-1 [21]. Moreover, PAR-1 can be activated by proteases other than FVIIa, thrombin and APC, including activated coagulation factor × (FXa), plasmin, trypsin, cathepsin G, elastase, chymase, and, as mentioned, MMP-1 [6,18], and multiple cell types present in the lung express PAR-1, including macrophages, mast cells, fibroblasts and airway smooth muscle cells [6]. Hence, the net effect of PAR-1 activation depends on the cell types and proteases present during various stages of the infection. This may also explain the partially contradictory results obtained on the role of PAR-1 in CLP-induced abdominal sepsis. Of note, however, in accordance with our current findings regarding lung pathology and neutrophil recruitment after infection with *S. pneumoniae*, PAR-1 was reported to participate in the acute lung inflammation elicited by intrapulmonary instillation of bleomycin, as reflected by reduced inflammatory cell influx in PAR-1 KO mice [22]. This [22] and other studies [23,24] have further implicated PAR-1 as a proinflammatory receptor in acute as well as chronic lung injury. It was therefore unexpected that PAR-1 KO mice displayed higher concentrations of the proinflammatory cytokines TNF-α, IL-6 and IFN-γ in lung tissue during pneumonia. We can only speculate on the mechanism: theoretically, the increase in cytokine generation in PAR-1 KO mice could be the result of diminished anti-inflammatory endothelial protein C receptor (EPCR-)APC effects via PAR-1 or to compensatory increases of other proinflammatory signaling pathways such as upregulation of PAR-2 to PAR-4 outweighing the loss of PAR-1 as a proinflammatory signaling pathway. Further studies are needed to dissect the exact mechanisms and cell types at play mediating PAR-1 effects after infection by *S. pneumoniae*.

## Conclusions

We show that in pneumococcal pneumonia, PAR-1 impairs the host defense response, as reflected by a reduced lethality, lower bacterial loads, lower lung histopathology scores and less pulmonary neutrophil influx in PAR-1 KO mice. Considering the complex role of PAR-1 in infection, related to the capacity of multiple proteases to activate PAR-1 resulting in differential cellular effects and the multiple cell types expressing PAR-1, this receptor at this moment does not represent a straightforward therapeutic target in severe pneumonia and sepsis.

## Key messages

• Protease activated receptor (PAR)-1 knock out (KO) mice have an improved survival as compared to wild-type (WT) mice in pneumococcal pneumonia.

• PAR-1 KO mice have lower bacterial loads in lungs and blood at 24 hours and in spleen and liver at 48 hours after induction of pneumococcal pneumonia as compared to WT mice.

• The favorable response in PAR-1 KO mice with regard to survival and bacterial outgrowth is accompanied by lower histopathology scores and less neutrophil influx in the lungs.

• Taken together, this study shows that PAR-1 hampers host defense in murine pneumococcal pneumonia.

## Abbreviations

APC: activated protein C; CAP: community-acquired pneumonia; CBA: cytometric bead array; CFU: colony forming units; CLP: cecal ligation and puncture; EPCR: endothelial protein C receptor; ELISA: enzyme-linked immunosorbent assay; FVIIa: activated coagulation factor VII; FXa: activated coagulation factor X; H & E: hematoxylin & eosin; IFN: interferon; IL: interleukin; KO: knockout; LD90: 90% lethal dose; mAb: monoclonal antibody; MCP: monocyte chemoattractant protein; MIP: macrophage inflammatory protein; MMP: matrix metalloproteinase; PAR: protease-activated receptor; PBS: phosphate-buffered saline; *S. pneumonia*: *Streptococcus pneumonia*; TNF: tumor necrosis factor; WT: wild type.

## Competing interests

The authors declare that they have no competing interests.

## Authors' contributions

MS participated in the design of the study, carried out the *in vivo *experiments and drafted the manuscript. CV participated in the design of the study, advised in laboratory matters and helped draft the manuscript. JJTHR performed pathology scoring, prepared part of the figures and helped draft the manuscript. ML helped to draft the manuscript and supervised the study. TP participated in the design of the study, supervised the study and helped draft the manuscript. All authors read and approved the final manuscript.
